# 4173 An interactive, online Research Education Hub built with a standard Learning Management System focused of education and career development for students, postdocs, faculty, and research staff

**DOI:** 10.1017/cts.2020.201

**Published:** 2020-07-29

**Authors:** Russell Lackey, Alfred Vitale, Edwin van Wijngaarden

**Affiliations:** 1 University of Rochester Medical Center

## Abstract

OBJECTIVES/GOALS: The University of Rochester CTSI Research Education Hub is designed to: 1) connect the local research community with essential internal and external educational resources; 2) create a community of inquiry and collaboration across the translational science workforce pipeline within the university. METHODS/STUDY POPULATION: The Research Education Hub (RE-Hub) utilizes the university’s widely used Learning Management System (LMS), Blackboard, and accessible to anyone at the university with a BlackBoard account. The RE-Hub greets users with an overview, an introduction of key local faculty experts in relevant research methodologies, and links to institutional research programs and helpdesks. Users are provided with curated educational resources organized by topic areas including, but not limited to, research methodology, statistical analysis, and grantsmanship. Discussion boards were created for users to ask general research questions and to connect with others in the translational research community. RESULTS/ANTICIPATED RESULTS: The RE-Hub was designed in Fall 2019 with the purpose of increasing utilization of university resources, including workshops, seminars, methods forums and consultation resources to improve translational science at the university. The RE-Hub was designed to be flexible and responsive to the changing needs of the local research community. User feedback will be used to identify improvements in the organization and content of the RE-Hub. Future improvements will include additional topic areas that span translational competencies, additional materials added to existing topic areas, and facilitation of better collaboration and integration of career development programs and grantsmanship resources. DISCUSSION/SIGNIFICANCE OF IMPACT: The Research Education Hub provides the University of Rochester translational science research community with a space to explore educational resources, to interact with colleagues and ask research related questions, and to help develop and/or improve other educational programs at the university.

